# Neural Differentiation of Human Pluripotent Stem Cells for Nontherapeutic Applications: Toxicology, Pharmacology, and *In Vitro* Disease Modeling

**DOI:** 10.1155/2015/105172

**Published:** 2015-05-25

**Authors:** May Shin Yap, Kavitha R. Nathan, Yin Yeo, Lee Wei Lim, Chit Laa Poh, Mark Richards, Wei Ling Lim, Iekhsan Othman, Boon Chin Heng

**Affiliations:** ^1^Department of Biological Sciences, Faculty of Science & Technology, Sunway University, No. 5 Jalan Universiti, 47500 Bandar Sunway, Selangor Darul Ehsan, Malaysia; ^2^Department of Physiology, The University of Hong Kong, Pokfulam, Hong Kong; ^3^School of Chemical & Life Sciences, Nanyang Polytechnic, 180 Ang Mo Kio Avenue 8, Singapore 569830; ^4^Jeffrey Cheah School of Medicine, Monash University Malaysia, Jalan Lagoon Selatan, 47500 Bandar Sunway, Selangor Darul Ehsan, Malaysia; ^5^Department of Biosystems Science & Engineering (D-BSSE), ETH-Zurich, Mattenstrasse 26, 4058 Basel, Switzerland

## Abstract

Human pluripotent stem cells (hPSCs) derived from either blastocyst stage embryos (hESCs) or reprogrammed somatic cells (iPSCs) can provide an abundant source of human neuronal lineages that were previously sourced from human cadavers, abortuses, and discarded surgical waste. In addition to the well-known potential therapeutic application of these cells in regenerative medicine, these are also various promising nontherapeutic applications in toxicological and pharmacological screening of neuroactive compounds, as well as for *in vitro* modeling of neurodegenerative and neurodevelopmental disorders. Compared to alternative research models based on laboratory animals and immortalized cancer-derived human neural cell lines, neuronal cells differentiated from hPSCs possess the advantages of species specificity together with genetic and physiological normality, which could more closely recapitulate *in vivo* conditions within the human central nervous system. This review critically examines the various potential nontherapeutic applications of hPSC-derived neuronal lineages and gives a brief overview of differentiation protocols utilized to generate these cells from hESCs and iPSCs.

## 1. Introduction

The term of human pluripotent stem cells (hPSCs) is an umbrella term that encompasses both human embryonic stem cells (hESCs) [1] and human induced pluripotent stem cells (hiPSCs) [2]. The distinct advantages that these cells have over adult stem cells are their unlimited proliferative capacity and much more extensive differentiation potential [1, 2]. As such, neural differentiation of PSCs could provide a promising cell source for regenerative medicine and cell therapy applications [2], for* in vitro* pharmacological and toxicological screening of neuroactive compounds [3, 4], as well as* in vitro* modeling of neurodegenerative and neurodevelopmental diseases [5].

Utilization of hESCs is ethically controversial because it involves the destruction of human embryos. Therefore, one way to circumvent these ethical issues is to reprogram somatic cells to iPSCs via recombinant expression of specific transcription factors such as OCT3/4, SOX2, KLF4, and c-MYC [6, 7]. Currently, it is generally accepted in the scientific community that hiPSCs are highly similar if not virtually identical to hESCs in terms of their morphology, surface marker expression, feeder dependence, and* in vivo* teratoma formation capacity [6, 8]. Various adult somatic cell types have been used to generate iPSCs such as hair follicle progenitors, bone marrow stromal cells, lymphocytes, skin biopsy, and even epithelial cells from the urinary tract [6, 9–12].

Because neural differentiation of hPSCs for therapeutic applications in regenerative medicine has already been extensively reviewed in the scientific literature, this review will give a brief overview of current protocols for neural differentiation of hPSCs followed by examination of the various nontherapeutic applications of hPSCs-derived neural lineages, in particular for* in vitro* toxicological and pharmacological screening of neuroactive compounds, as well as for* in vitro* modeling of neurodegenerative and neurodevelopmental diseases.

## 2. Neural Differentiation of hPSCs: Progress towards a Defined Culture Milieu and Small Molecule Inducers

The unlimited self-renewal capacity and proliferative potential of hPSCs offer great promise to both basic research and translational clinical applications as an inexhaustible and replenishable cell source. Nevertheless at the undifferentiated pluripotent stage, these cells cannot be deployed directly into patients due to their tumorigenic potential [13]. Thus, establishment of efficient stepwise differentiation protocols for directing hPSCs into specific cell lineages is an essential prerequisite for both therapeutic and basic research applications.

Previously, two conventional techniques commonly utilized to initiate neural differentiation of hPSCs are embryoid body (EB) formation (from dissociated suspension culture) and cocultivation with stromal cell lines [14]. However, these approaches are complicated, time consuming, and ineffective, yielding great variability in results. Alternative commercial good manufacturing practice- (GMP-) compliant neural induction mediums and kits are emerging despite the high costs. Significant progress has been made in developing protocols for efficient neural differentiation of hPSCs, and the current trend gravitates towards chemically defined culture milieu supplemented with small molecule inducers of neural differentiation. The use of small molecules is highly desirable because it is cost effective and stable and reduces experimental variability [15]. Moreover, the biological effects of small molecules are rapid, reversible, and tuneable by varying the concentration or duration of exposure. Many studies have demonstrated the significant benefits of a small molecule-based system for stem cell differentiation. We therefore emphasize here recent strategies using small molecules for neural induction of hPSCs.

Neural differentiation of hPSCs generally relies on the interplay of activation and inhibition of multiple developmental signalling pathways tightly controlled by growth factors, cytokines, and epigenetics mechanisms. Improved understanding of developmental signalling pathways has guided the design of neural differentiation protocols. An increasing number of studies have illustrated the use of small molecules to modulate the key developmental signalling pathways known to regulate neural differentiation of hPSCs. Chambers and colleagues demonstrated that the combined use of multiple small molecule signalling pathway inhibitors, including LDN 193189, SB 431542, SU 5402, CHIR99021, and DAPT could accelerate the neural differentiation of hPSCs [16]. Neely et al. [17] showed that a newly developed highly selective small molecule BMP-inhibitor, DMH-1, can effectively induce neurogenesis of hiPSCs when combined with SB 431542. DMH-1 is postulated to substitute the function of commonly used but expensive growth factors. The same study also highlighted that optimal small molecule concentration is crucial for appropriate expression levels and timing of activation of various transcription factors related to neuronal lineage specification. Another study illustrated a highly efficient protocol for directing monolayer-cultured hESCs into homogenous primitive neural stem cells (pNSCs) with combined inhibition of the GSK3, TGF-*β*, and Notch signalling pathways [18]. Long-term expansion culture of pNSCs with a cocktail of leukaemia inhibitory factor (LIF), CHIR 99021, and SB 431542 retained remarkably high neurogenic propensity and plasticity [18]. The same study also revealed that the addition of a *γ*-secretase inhibitor (compound E) induced rapid neural differentiation. Such an approach enables controlled expansion of the desired neural precursor populations and overcomes the limitations of the directed differentiation process, which often yields heterogeneous neural populations.

More recently, studies on small molecule inducers of neuronal differentiation have focused on directing hPSCs into each of the four major specific neuronal sublineages, that is, dopaminergic, serotonergic, GABAergic, and cholinergic/motor neurons. This is because many neurodegenerative diseases are characterized by the dysfunction or loss of specific neuronal sublineages. For example, Parkinson's disease is characterized by dysfunction/loss of dopaminergic neurons [19, 20], while Alzheimer's disease on the other hand is thought to arise from the dysfunction/loss of cholinergic neurons [21]. It is well known that Huntington's disease is characterized by the degeneration of GABAergic medium-sized spiny neurons (MSNs) [22], while amyotrophic lateral sclerosis is characterized by the degeneration of cholinergic motor neurons [23]. Hence, there is much interest in developing small-molecule based differentiation protocols for deriving specific neuronal sublineages from hPSCs (Table 1), for* in vitro* modeling of neurodegenerative diseases, as well as for pharmacological screening of new drugs to treat these diseases.

Chambers and colleagues developed a protocol for differentiation of hPSCs into dopaminergic neurons, using dual inhibition of SMAD signals [24]. This was based on previous knowledge that the endogenous bone morphogenic protein (BMP) antagonist, noggin, is a critical neural-inducer in frog [31, 32] and that inhibition of Activin/Nodal/TGF*β* signalling by the small molecule SB 431542 (Activin A receptor-like kinase ALK4, 5, 7 inhibitor) has been shown to enhance neural induction of hESCs [33]. This dual-SMAD inhibition protocol bypassed EB formation and yielded over 80% neural induction efficiency [24]. The study of Kriks et al. [25] also utilized a dual SMAD inhibition protocol to derive dopaminergic neurons from human embryonic stem cells, by utilizing the small molecules purmorphamine and CHIR99021, together with FGF8 and recombinant sonic hedgehog (SHH C25II). In another study by Mak et al. [26], dopaminergic neurons were derived from six hiPSCs lines of patients with Parkinson's disease, utilizing dorsomorphin that had BMP-antagonistic activities similar to noggin. Neural precursors were derived with a 5-stage embryoid body differentiation protocol, using a combination of dorsomorphin and SB431542, with neuronal maturation achieved by substituting sonic hedgehog (SHH) with purmorphamine or smoothened agonist.

To date, the only reported study on the use of small molecules for directing hPSCs differentiation into GABAergic neurons involved combined transient stimulation of muscarinic and GluR1 receptors with their respective small molecule agonists [27]. Although there was inhibition of overall neurogenesis, enhanced differentiation into immature GABAergic neurons was observed. Other studies utilizing protein-based growth factors have reported that Activin A [34], Sonic Hedgehog (SHH), and DKK1 protein [35, 36] could drive PSCs differentiation into GABAergic neurons. It is probable that, in the future, these protein-based growth factors could be substituted with small molecules that activate similar signaling pathways.

Currently, there have yet been no reported studies on small molecule induction of serotonergic neurons from PSCs. However, noggin has been reported to enhance mouse embryonic stem cell differentiation into serotonergic neurons [37, 38]. It is possible that small molecules eliciting similar signalling pathways as noggin could enhance hPSCs differentiation into serotonergic neurons.

A number of studies have reported that retinoic acid in combination with purmorphamine that activates the sonic hedgehog signaling pathway could induce hPSCs differentiation into cholinergic motor neurons [28, 29]. Amoroso and colleagues [30] further reported that a third small molecule, SMO agonist SAG, could also be used in combination with retinoic acid and purmorphamine for induction of cholinergic motor neurons from PSCs. In another study on mouse embryonic stem cells by Wang et al. [39], it was reported that the small molecule icaritin could also induce hPSCs differentiation into cholinergic motor neurons, but this has yet to be tested on hPSCs.

In conclusion, synthetic small molecules are powerful tools for manipulation of stem cell lineage fate and differentiation pathways, and they also provide deep insights into the underlying molecular mechanisms that control stem cell fate. Nonetheless, more potent and specific small molecules are required for precise control of neurogenesis. Continuous characterization of small molecules and comprehensive understanding of the underlying molecular mechanisms are necessary for achieving greater efficiency in the neural differentiation of hPSCs. More recently, Wen and Jin [40] developed a robust protocol that efficiently yields NSCs from hPSCs without utilising either small molecules or the EB formation step. The generated NSCs retained the same differentiation capacity as that derived by the EB formation approach. It was hypothesized that a dynamic change of cell-substrate matrix interactions via a short suspension culture led to ectoderm lineage specification by hPSCs, even though the underlying mechanisms remain uncharacterized.

## 3. Nontherapeutic Applications of Human Pluripotent Stem Cells-Derived Neural Lineages

Neuroscience research has advanced at a rapid pace over the past few decades, yet it remains as one of the greatest challenges in the 21st century. The functioning of the human brain remains enigmatic and very few research discoveries have been translated into the development of innovative treatment for neurological disorders and diseases, despite the best collaborative efforts of academic research institutions and the biopharmaceutical industry [41].

This could be because our understanding of neurodevelopment and neurological disease pathophysiology has been severely hampered by the relative inaccessibility of human brain tissues. It is often difficult to obtain neural tissue samples from a live human brain for disease modeling, molecular analysis, and drug screening. Until recently, most of the published data in neuroscience research was based on animal models, transformed cell lines, and tissues from aborted foetuses or discarded poor-quality pathological samples from brain surgery patients [42]. Although these have undoubtedly made valuable contributions in deciphering neurodevelopmental mechanisms, as well as in providing insights into the pathogenesis of neurological disorders and the roles of specific genes in neurodegeneration, it must nevertheless be noted that the experimental data obtained from animals and transformed cell lines are often not representative of human neurological function due to greater complexity of the human brain and interspecies physiological differences. In addition, neurons from different species may exhibit different electrophysiological properties [43], and neural tissues sourced from foetuses or surgery are extremely rare. Given these limitations, there is a dire need for alternative sources of human neural tissues that are readily available and physiologically relevant.

The discovery of both hESCs and hiPSCs could provide new tools to study neural development and neurological diseases mechanisms* in vitro*, as well as for screening and development of new drugs for neurological disorders. Neuronal lineages derived from hPSCs provide distinct advantages over other model systems as they are more representative of human neural physiology and can accurately recapitulate the* in vivo* conditions of neurodegenerative diseases* in vitro*, hence overcoming the problem of species specificity. This in turn has led to increased adoption of hPSCs models by neuroscience researchers for pathological modeling, toxicology assessment, and drug screening. In addition, the biopharmaceutical industry has begun to embrace the human pluripotent stem cell-based approach for drug development and predictive toxicology assays [44].

## 4. Toxicology

The human body is constantly being exposed to potential neurotoxic compounds in daily life, ranging from contaminating pesticides on vegetables to the veritable menagerie of industrial chemicals found in human consumer products. A large number of these pose serious health hazards to the human nervous system, yet little is known about how exposure to compounds will impact human neural function and development. There is a dire lack of neurotoxicity data on the various compounds that we are being exposed on a daily basis. The developing human brain is particularly susceptible to environmental toxicants, and the damage induced by neurotoxins can range from onset of neurodevelopment disorders to long-lasting neurological impairments [45]. With growing awareness and concern regarding the potential neurotoxicity of environmental contaminants, prescription drugs, and industrial chemicals, much attention and effort has been directed towards neurotoxicology.

Neurotoxicology refers to the study of the adverse effects of chemical, biological, and physical factors on neural function and development together with their underlying mechanisms. Adverse effects can encompass interference with normal functioning of the nervous system, morphological changes such as neuronopathy (loss of neurons) or axonopathy (degeneration of nerve axon), and neurochemical changes [46]. Currently, the overwhelming majority of neurotoxicity studies reported in the scientific literature rely on either* in vivo* animal models or immortalized cell lines as* in vitro* models. Experimental data from animal-based studies are often difficult to interpret due to interspecies differences in neuroanatomy and neural physiology. For example, Thalidomide [47, 48] and 13-*cis-*retinoic acid are well-known human teratogens, but are innocuous in murine models [49, 50]. Additionally, animal-based neurotoxicity screening is labor-intensive, expensive, and time consuming, in addition to being ethically contentious. While immortalized human neural cell lines are indeed useful for* in vitro* neurotoxicity screening, these cell lines derived from tumours invariably contain chromosomal and genetic aberrations and may not exhibit the same phenotype as primary cultures of normal human neurons, astrocytes, and oligodendrocytes [51].

In response to current legislative framework such as the Registration, Evaluation, and Authorization of Chemicals (REACH) in the European Union [52] and the need for alternative screening tests with higher reliability and effectiveness to characterize the neurotoxic potential of compounds without the use of live animals, efforts are being directed towards the development of human pluripotent stem cell-based assays [53, 54]. The application of hPSCs and their neural derivatives in neurotoxicology assays has many advantages over other* in vitro* models, due to the unlimited proliferative potential of these cells, as well as their ability to recapitulate various important neurodevelopmental encompassing proliferation, migration, and differentiation [55].

The embryonic stem cell test (EST) is an existing validated approach designed by the European Centre for the validation of alternative methods (ECVAM) to screen compounds for developmental neurotoxicity [56]. Although this murine-based EST has proven to be reliable [57], in order to obviate confusion in data interpretation associated with interspecies differences, Adler and colleagues [58, 59] pioneered a humanized EST and showed the necessity for a human-based system to detect human-relevant toxicants by demonstrating that some chemicals display species-specific cytotoxic effects. For example, 13-*cis* retinoic acid is cytotoxic to humans but not to mice. Moreover, they illustrated that marker gene expression analysis may a useful endpoint for developmental toxicity screening studies. Currently, there are only a few studies that have focused specifically on the use of hPSCs in developmental neurotoxicity. Stummann and colleagues [60] developed a neurodevelopmental toxicity assay based on two-step differentiation of hESCs into neural stem cells, followed by mature neurons. They found that a previously EST misclassified heavy metal methylmercury (MeHg) compound could disrupt the early stages of neural differentiation of hESCs. This thus underlined the importance of utilizing physiologically relevant species-specific toxicology screening models [60].

Neural stem cells (NSCs) or neural progenitor cells (NPCs) derived from hPSCs have much potential in the development of neurotoxicity screening assays. NSCs are particularly useful for studying chemical interference in neural differentiation as they are present within a range of developmental stages of the nervous system and are more highly sensitive to neurotoxic perturbations, as compared to other neural lineages [61]. The presence of NSCs in the discrete neurogenic area of adult brain [62] would make these cells suited for neurotoxicity studies in the adult nervous system. In the presence of epidermal growth factor, NSCs can develop into free-floating neurospheres, comprising a heterogeneous mixture of cell lineages. Neurospheres have been utilised to assess developmental neurotoxicity as these can recapitulate key processes of human neurodevelopment, including proliferation, differentiation, and migration* in vitro* [63]. Previous studies have analyzed how neurospheres are affected by exposure to neurotoxicants by assessing a range of endpoints such as viability, proliferation, differentiation, and migration. For example, the exposure of neurospheres to methylmercury chloride and mercury chloride led to less differentiation into neural lineages, as well as inhibited cell migration [63], while exposure to polybrominated diphenyl ether (PBDE) had negligible cytotoxic effects but significantly decreased cell migration and neural differentiation during brain development [64]. Exposure to MeHg also resulted in significant detrimental effects on neuronal differentiation [61].

More recently, the advent of induced pluripotent stem cell (iPSC) technology provides new opportunities for neurotoxicology assessment. Utilizing human iPSCs for neurotoxicology screening assays not only circumvents ethical issues in the use of pluripotent embryonic stem cells derived from culled human embryos, but also enables personalised screening for predicting individual susceptibility to various environmental toxicants. There is much evidence in the scientific literature, which has shown that variation in environmental and genetic risk factors between individuals may predispose people to different susceptibility to environmental toxicants [65]. iPSC lines generated from a wide range of human individuals with well-characterised adult phenotypes can serve as model systems for understanding how variation in toxic susceptibility displayed by different individuals correlates with their genetics, disease state, and other observable phenotypes [51]. iPSC lines isolated from patients with neurological diseases may also help us to understand the relationship between neurotoxicants and neurological diseases. Disease-specific iPSC lines allow scientists to examine progression of neurological diseases and how changes in neural function elicited by neurotoxicants could be influenced by underlying genetic inheritance [66].

## 5. Utilizing Stem Cell Derived Neurons for Pharmacological Screening and Drug Development

Neurological and neurodegenerative diseases present poor prognosis and significant clinical challenges, despite the tremendous amount of money and effort that have been invested in drug development research for the treatment of these diseases. Current treatment modalities for neurodegenerative diseases are mostly palliative in nature, resulting in symptomatic relief rather than alteration of disease prognosis, and there is a dearth of effective medications that slow, prevent, or reverse the progression of neurodegenerative diseases thus far. Available medications for Alzheimer's disease and Parkinson's disease can only alleviate the symptoms, yet they are plagued by tolerability issues. The prognoses for Huntington's disease and amyotrophic lateral sclerosis are even bleaker.

A sizable proportion of the ageing population is afflicted with neurodegenerative diseases and is responsible for the rising share of morbidity and mortality associated with the rapidly ageing populations of both developed and developing countries worldwide. The economic burden of these diseases is massive, and the human and social costs of caring for these patients are incalculable. As the global elderly population is predicted to triple by 2050 [67], the challenges that neurodegenerative diseases pose to our society will only increase. Hence, there is an urgent need for new and effective treatment modalities for neurological diseases.

Drug development is extremely costly and challenging. The estimated expenditure for a newly developed drug to reach the market is approximately US $1.5 billion, and this is expected to continue to skyrocket in the near future [68]. High rates of late-stage attrition and withdrawals of candidate drugs have imposed a multi-billion dollar burden on pharmaceutical companies over the past few decades and this remains one of the most pressing challenges in the biopharmaceutical industry [69]. Low efficacy and safety concerns are the major factors contributing to 90% of lead compound failures in clinical trials. This is not surprising in view of the fact that most candidate drug compounds are only tested on relevant patients in clinical trials. Conventional drug development has relied heavily on animal-based systems as human surrogates to evaluate toxicity and efficacy of candidate drug compounds prior to commencement of clinical trials. Positive preclinical results observed in animal models are not necessarily recapitulated in humans owing to discrepancies in disease mechanisms and physiology between animals and humans. The uncertainty of drug translatability to humans often leads to unexpected failure upon entering the clinical trial stages, making the drug development project intractable. In addition, other possible reasons for the failure of translation from the laboratory bench to clinic in the development of drugs targeting neurodegenerative diseases include poor drug penetration due to blood-brain barrier, problems associated with pharmacokinetics, poor patient selection criteria, and incorrect end points for measuring drug effects [41].

Pharmaceutical companies and drug regulatory authorities have been seeking to establish better predicative models to minimize attrition of candidate drugs at the clinical trial stages, through early identification of toxicity and adverse effects of a candidate drug compound, before lengthy and expensive clinical trials are carried out. It was reported that, for every 1% increase in predictability of the toxicity of drug candidate compounds to the human body, a pharmaceutical company could save up to US $100 million [70]. The emergence of hPSC technology offers access to difficult-to-obtain human neural cell populations that are more physiologically and pharmacologically relevant for drug screening. Additionally, hPSC-derived neurons can overcome many other pertinent challenges faced in drug development by providing an unlimited and consistent cell source for high-throughput screening, thereby saving costs and time, as well as allaying ethical concerns associated with animal testing. Furthermore,* in vitro* screening assays based on hPSC-derived neurons can eliminate patient risk issues and allow the study of drug effect on multiple cell types derived from the human body, which could eventually increase the success rates of subsequent clinical trials [71].

Many studies have reported the use of hiPSCs-derived neural cells for the drug screening process (Table 2). Yahata et al. [72] generated hiPSCs-derived amyloid *β*-secreting neurons expressing functional *β*- and *γ*-secretase and utilized these cells to screen for novel effective anti-amyloid *β* drugs against Alzheimer's disease. Moreover, disease-specific hiPSCs are useful in assessing the effects of novel therapeutic compounds, resulting in a greater likelihood of positive efficacy in humans. For example, Brennand et al. [73] reprogrammed fibroblasts from Schizophrenia (SCZD) patients into hiPSCs and subsequently differentiated these into neurons. These hiPSCs-derived neurons express disease-specific phenotypes, including decreased neuronal connectivity and differential gene expression patterns in the cAMP and WNT signalling pathways. It was observed that the neuronal activity of SCZD neurons was improved following treatment with the antipsychotic drug Loxapine. Another study demonstrated that application of valproic acid (VPA) and tobramycin in a spinal muscular atrophy (SMA) neuron model established from hiPSCs could upregulate expression of survival motor neuron (SMN) protein [74]. In addition, Yagi et al. [75] have identified a promising drug candidate for Alzheimer's disease therapy by screening with hPSC-derived neurons. The aberrantly increased amyloid *β*42 secretion attributed to mutant presenilin in AD-iPSC-derived neurons was reduced by treating with compound W (*γ*-secretase inhibitor and modulator), indicating the potential of compound W as a therapeutic drug [75].

Considering the available evidence in the scientific literature, we can conclude that hPSC technology indeed provides a powerful tool for pharmacological screening and drug development, bridging the translational gap from the laboratory to clinical trials, as well as presenting an excellent and cost effective* in vitro* drug screening system to overcome current challenges faced by the biopharmaceutical industry. Furthermore, iPSC technology could be applied to disease- and patient-specific studies to elucidate individual variations in pharmacokinetics and pharmacodynamics, thereby facilitating the development of personalised medicine [76].

Although promising, there are many hurdles to be overcome in the application of hPSC technology in pharmacological assays. The growth and expansion of hPSCs are technically demanding and require greater expertise than other commonly utilized cell types for* in vitro* screening assays. For example, the culture of hPSCs requires constant monitoring and manual purging of spontaneously differentiated cells. Additionally, it is necessary to assess iPSC clones from the same patient for consistent genotypes and phenotypes, as they may acquire undesirable genetic and epigenetic alterations during the reprogramming process [77–79]. Their propensity to differentiate into various lineages needs to be analysed, as adult somatic cell-derived iPSCs may preserve some degree of epigenetic memory of their past and therefore exhibit biased differentiation propensity to their somatic lineage of origin [80, 81]. For* in vitro* screening assays, it is imperative that an optimized differentiation protocol for hPSCs developed to derive a population of homogeneous and stable neural cells. Rigorous characterisation of intermediate and terminally differentiated populations is essential for the establishment of highly specialised cellular models with better reproducibility and consistency, which could serve as a high-predictivity drug screening tool. Given the ongoing intensive efforts of research laboratories and pharmaceutical industries to establish safer and more efficient protocols in hPSC technology, there is cause for optimism that hPSC technology will be applied broadly in the drug development pipeline in the near future and may ultimately contribute to the discovery of new cures for neurological diseases with currently poor clinical prognosis.

## 6. *In Vitro* Modeling of Neurodegenerative and Neurodevelopmental Diseases

As mentioned previously, animal models to study neurological diseases have inherent limitations and disadvantages and do not fully recapitulate the human neural phenotype. Hence, iPSCs may provide a vital tool for the study of human neurodegenerative and neurodevelopmental diseases (Table 3) through their differentiation into functional neurons in a controlled* in vitro* environment [82]. Neurodegeneration refers to the gradual deterioration of cognitive abilities due to structural changes that prevents neurons from functioning normally [83], whereas neurodevelopmental disorders are characterized by impairment of neuronal function during brain development which adversely affects emotions, self-control, learning ability, and memory of an individual.

Amongst the most notable and frequently occurring neurodegenerative diseases are Alzheimer's disease, Parkinson's disease, Huntington's disease, and amyotrophic lateral sclerosis [83]. Typically, these diseases are characterized by being age-related [83] meaning that neurons gradually lose their function as these neurodegenerative diseases progress with age. Currently, reprogramming technology allows researchers to study the development and progression of neurodegeneration in a human system and this may in turn enables the development of new early diagnostic technologies and improved treatment modalities.

Alzheimer's disease is the most common type of age-related dementia found in aging population and is the 6th leading cause of death in USA [100]. Clinically, the disease is characterized by progressive memory loss and decline in cognitive abilities [101]. Alzheimer's disease can be caused by mutations in presenilin- (PS-) 1, PS2, or amyloid precursor protein (APP), as well as microtubule-associated protein tau [102]. These mutations are inherited in an autosomal dominant manner and are thus referred to as familial Alzheimer's disease [102]. Israel and colleagues [84] demonstrated that iPSC-derived neurons from patients with APP duplications have elevated amyloid *β*40 and patients with sporadic Alzheimer's disease can have increased amyloid *β*40 expression and enlarged endosomes [84]. Additionally, they also observed increased expression of phosphorylated Tau, the precursor to neurofibrillary tangle formation, besides elevated amyloid *β*.

Huntington's disease is another progressive neurodegenerative disorder. It is caused by an elongated stretch of a triplet-repeat CAG (coding for glutamine) within the huntingtin gene on chromosome 4 [103]. This repeating of CAG results in a polyglutamine domain [104]. Because it is caused by mutations in a single gene, several groups have attempted to model Huntington's disease with iPSCs derived from Huntington's disease patients [85, 86]. An et al. [85] reported that neural stem cells derived from Huntington's disease hiPSCs were more susceptible to oxidative stress than normal iPSCs. This was overcome when Huntington's disease iPSC CAG repeat was corrected by genetic engineering [85]. Huntington's disease consortium reported that NSCs with higher CAG repeat failed to develop into functional neurons and gradually died off, unlike NSCs with lower CAG repeats [86]. In addition, distinct morphological changes associated with neural progenitor cells (NPC) derived from Huntington's disease iPSC lines were also observed [86].

Amyotrophic lateral sclerosis is a progressive fatal disease that basically affects both upper and lower motor neurons, resulting in dysfunction and death of the affected neurons [105]. Amyotrophic lateral sclerosis is caused by mutations in superoxide dismutase 1 (SOD1) enzyme [106] and vamp-associated protein B/C (VAPB) [107]. Mitne-Neto et al. [87] studied iPSCs lines from amyotrophic lateral sclerosis patients with mutations on the VAPB gene as well as from their unaffected siblings as controls. They showed a significant reduction in VAPB protein levels in amyotrophic lateral sclerosis iPSC-derived motor neurons but could not detect any cytoplasmic aggregation [87].

In neurodevelopmental disorders, iPSCs-based models can recapitulate the early steps of neuronal differentiation known to result in these pathologies and can facilitate the study of the cellular and molecular causes of these disorders. Recently, several groups have used hPSCs to model other less common neurodevelopmental disorders such as autism spectrum disorder (ASD) [88], schizophrenia (SCZD) [89, 90], Rett syndrome (RTT) [91–94], fragile X syndrome [95–97], and Timothy syndrome [98, 99].

Several studies have thus shown that iPSCs technology has much potential for investigating the molecular mechanisms of an array of neurological diseases that currently have no cure. Modeling neurodegenerative and neurodevelopmental diseases using this technology can possibly have a significant impact on the development of new therapeutic modalities for these diseases.

## 7. Conclusion and Future Outlook

Despite some lingering doubts that iPSCs are not exactly identical to hESCs [108], iPSC technology has largely circumvented ethical obstacles previously associated with hESC research and is gradually supplanting hESC as an important tool for* in vitro* modeling of human neurological diseases and developing drug screening systems, identifying therapeutic targets, in addition to providing autologous cell sources for therapy of various neurological diseases [76, 82, 109–111]. A promising nontherapeutic application of PSCs that has yet to be developed is for* in vitro* study and modeling of infectious diseases that specifically target the nervous system such as Nipah virus, Japanese encephalitis, and Rabies [112]. This is anticipated to be realized in the near future.

## Figures and Tables

**Table 1:** Small-molecule based culture protocols for inducing hPSCs differentiation into specific neuronal sublineages.

Neuronal sublineages	Key references	Small molecules utilized
Dopaminergic	Chambers et al. [24]	SB431542 (together with the protein noggin)
	Kriks et al. [25]	Purmorphamine + CHIR99021 (together with the growth factor FGF8 and recombinant sonic hedgehog)
	Mak et al. [26]	Dorsomorphin + SB431542

GABAergic	Samarasinghe et al. [27]	Agonists of muscarinic and GluR1 receptor

Cholinergic motor neurons	Li et al. [28]	Retinoic acid + purmorphamine
	Hu and Zhang [29]	Retinoic acid + purmorphamine
	Amoroso et al. [30]	Retinoic acid + purmorphamine + SMO agonist SAG

**Table 2:** Examples of drug leads screened with hPSCs-derived neuronal lineages.

Drug lead	Disease model	Key references
Sulindac sulfide Compound E *β*-secretase inhibitor IV	Alzheimer's disease	Ebert et al. [74]

Compound W	Alzheimer's disease	Yagi et al. [75]

Loxapine	Schizophrenia	Brennand et al. [73]

Valproic acid	Spinal muscular atrophy	Ebert et al. [74]

**Table 3:** *In vitro* modeling of neurodegenerative and neurodevelopmental disorders with hPSCs-derived neuronal lineages.

Disease model	Key references
Alzheimer's disease	Israel et al. [84]

Huntington's disease	An et al. [85] The HD iPSC Consortium [86]

Amyotrophic lateral sclerosis	Mitne-Neto et al. [87]

Autism spectrum disorder	Kim et al. [88]

Schizophrenia	Chiang et al. [89] Brennand et al. [90]

Rett syndrome	Ananiev et al. [91] Cheung et al. [92] Li et al. [93] Williams et al. [94]

Fragile X syndrome	Sheridan et al. [95] Bar-Nur et al. [96] Liu et al. [97]

Timothy syndrome	Paşca et al. [98] Krey et al. [99]
